# Out of the silos: identifying cross-cutting features of health-related stigma to advance measurement and intervention

**DOI:** 10.1186/s12916-018-1245-x

**Published:** 2019-02-15

**Authors:** Wim H. van Brakel, Janine Cataldo, Sandeep Grover, Brandon A. Kohrt, Laura Nyblade, Melissa Stockton, Edwin Wouters, Lawrence H. Yang

**Affiliations:** 1 Amsterdam, Netherlands; 2 Department of Physiological Nursing, Center for Tobacco Control Research and Education, University of California San Francisco, 2 Koret Way, San Francisco, CA 94143-0610 USA; 3 Department of Psychiatry, PGIMER, Chandigarh, India; 4 Department of Psychiatry and Behavioral Sciences, George Washington University, Washington, DC USA; 5 RTI International, Washington, DC USA; 6 Epidemiology Department, UNC Gillings School of Global Public Health, Chapel Hill, NC USA; 7 Centre for Longitudinal & Life Course Studies, University of Antwerp, Antwerp, Belgium; 8 Centre for Health Systems Research & Development, University of the Free State, Bloemfontein, South Africa; 9 College of Global Public Health, New York University, New York, NY USA; 10 Mailman School of Public Health, Columbia University, New York, NY USA

**Keywords:** Health-related stigma, measurement, interventions, cross-cutting approaches, HIV, mental health, leprosy, tuberculosis, neglected tropical diseases, disabilities

## Abstract

**Background:**

Many health conditions perceived to be contagious, dangerous or incurable, or resulting in clearly visible signs, share a common attribute – an association with stigma and discrimination. While the etiology of stigma may differ between conditions and, sometimes, cultural settings, the manifestations and psychosocial consequences of stigma and discrimination are remarkably similar. However, the vast majority of studies measuring stigma or addressing stigma through interventions employ a disease-specific approach.

**Main body:**

The current paper opposes this siloed approach and advocates a generic concept of ‘health-related stigma’ in both stigma measurement and stigma interventions. Employing a conceptual model adapted from Weiss, the current paper demonstrates the commonalities among several major stigmatized conditions by examining how several stigma measurement instruments, such as the Social Distance Scale, Explanatory Model Interview Catalogue, Internalized Stigma of Mental Illness, and Berger stigma scale, and stigma reduction interventions, such as information-based approaches, contact with affected persons, (peer) counselling, and skills building and empowerment, were used successfully across a variety of conditions to measure or address stigma. The results demonstrate that ‘health-related stigma’ is a viable concept with clearly identifiable characteristics that are similar across a variety of stigmatized health conditions in very diverse cultures.

**Conclusion:**

A more generic approach to the study of health-related stigma opens up important practical opportunities – cross-cutting measurement and intervention tools are resource saving and easier to use for personnel working with multiple conditions, allow for comparison between conditions, and recognize the intersectionality of many types of stigma. Further research is needed to build additional evidence demonstrating the advantages and effectiveness of cross-condition approaches to stigma measurement and interventions.

## Background

Many health conditions perceived to be contagious, dangerous or incurable, to result in clearly visible signs, or to be caused by breaking taboos or immoral behavior share a common attribute – an association with stigma and discrimination. These health conditions are diverse in nature and include infectious diseases like HIV, tuberculosis (TB), leprosy and lymphatic filariasis, non-infectious chronic conditions such as epilepsy and cancers, and mental health conditions such as schizophrenia, depression, and substance abuse. Jones et al. [1] proposed six features, namely, (1) esthetics, (2) concealability, (3) course, (4) disruptiveness, (5) origin, and (6) peril, that help in recognizing and understanding why particular conditions are more vulnerable to health-related stigma, what factors would worsen or reduce a given stigma, and why some stigmas may be easier to address than others.

People often have co-morbidities and live with one or more of these health conditions and experience simultaneously different types of health-related stigma. Stigma is problematic because it affects people psychologically and restricts their social participation, and it can also create barriers to accessing healthcare, including retention in care for people living with HIV (PLHIV), relationships, education, and housing, thereby further marginalizing already vulnerable populations [2–4]. While the etiology of stigma may differ between conditions and, sometimes, cultural settings, the manifestations and psychosocial consequences of stigma and discrimination are remarkably similar [3, 5, 6]. Regardless of the condition, stigma is a dynamic process enacted through structures and individuals, mediated by relationships of power and control that are constantly being produced and reproduced [7]. Similarities across conditions are most likely due to the fact that the core of stigma is social in nature and therefore a common problem based on common human interpersonal responses to differentness and the mechanisms by which these responses might be expressed [8, 9]. Nevertheless, responses to persons with the same condition may also differ in different locations, based on local differences in social determinants of stigma (e.g., religious beliefs). They may vary between conditions, depending on perceived cause and danger (e.g., in HIV or leprosy, people might avoid sharing a meal to avoid infection).

The cross-cutting nature of stigma is evidenced by the measurement methods used and the interventions that have been shown to be effective to reduce stigma or mitigate its impact across conditions [3, 10–13]. In many of the disciplines dealing with stigmatized conditions, the problem has been recognized and is addressed to some extent, but often only in a condition-specific manner. One challenge is that the funding, research, assessment tools, and interventions often address stigma related to only one particular condition. If measurement tools and interventions that assess and address common dimensions of stigma were possible, the scarce resources to address stigma could be used more efficiently and healthcare providers could use the same tools and approaches, across conditions. Several theoretical models describing common elements of stigma have been proposed, including those by Scambler [14, 15], Link and Phelan [16], Pescosolido et al. [17], and Weiss [5].

### Health-related stigma

Stigma has been extensively studied in leprosy, mental health, HIV, epilepsy, and physical disability [3]. Lung cancer can also conjure a similar attribution of blame as that found with HIV and/or AIDS due to its frequent association with smoking cigarettes (tobacco) [18]. Yet, most of these have been studied only within their own field, often with development of condition-specific measurement instruments and interventions. From a health systems perspective, the application of generic tools for stigma assessment and of the same or similar interventions to address multiple stigmas would be highly beneficial. This benefit becomes even more evident in the light of an increasing frequency of co-morbidities and of the compounding impact of multiple intersecting stigmas.

To address this ‘siloed approach’ to stigma, the concept of ‘health-related stigma’ has been advocated [19, 20]. It should be noted that discrimination, also known as enacted or experienced stigma, is part of the construct of stigma. Health-related stigma is a personal experience related to a health condition [21], characterized by the perception of exclusion, rejection, and blame [22], and contributes to psychological, physical, and social morbidity [23]. The judgment inherent in any health-related stigma is medically unwarranted and may adversely affect health status and health outcomes [22]. Health-related stigma is associated with depression and limited social support and acts as a barrier to healthcare access, treatment uptake, retention, and adherence [3, 24–31]. It thus contributes to increased severity of morbidity and disability [32, 33], prolonged treatment duration and, through poor adherence, to development of drug resistance [34]. For example, stigma among individuals with mental illness can lead to adverse coping behaviors, including secrecy and withdrawal from others who do not share the stigmatizing status [35, 36], and has shown negative impact on treatment seeking (showing consistent small-to-moderate negative effects in a meta-synthesis [37]). In the field of HIV, stigma hinders access to and engagement in the HIV care continuum as a barrier to HIV testing, linkage to care, retention, and treatment adherence, and detrimentally impacts mental and physical wellbeing [30, 38, 39]. However, with the exception of several literature reviews on stigma measurement and interventions [3, 10–12, 40], there is a gap in evidence in the published literature demonstrating the case for a cross-cutting approach to reduction and mitigation of the intrapersonal and interpersonal aspects of stigma. This paper seeks to address this gap using research data of studies on stigma and discrimination related to a number of diverse conditions.

### Conceptual model

**Fig. 1:**
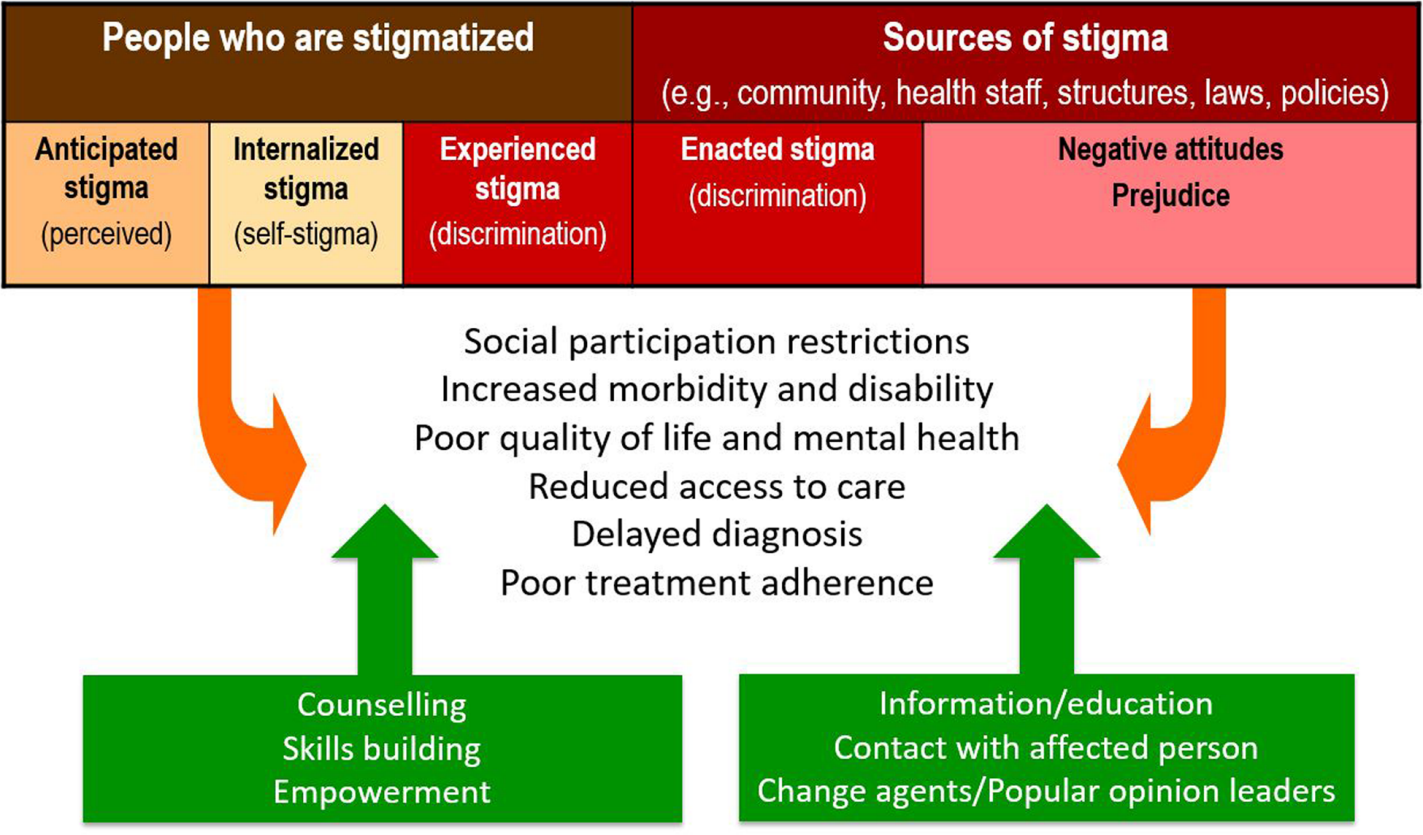
An assessment and intervention model of health-related stigma (model modified from Weiss [5])

For this paper, we will use a conceptual model (see Fig. 1), which is both a simplification and an expansion of the model proposed by Weiss [5], which in turn was an extension of Scambler’s Hidden Distress Model [14]. This model differentiates two main perspectives on health-related stigma, that of persons who are being stigmatized, and that of ‘those who stigmatize’. We have called the latter ‘sources of stigma’ to allow inclusion of structural forms of stigma. It is important to realize that people may belong to both categories. For example, persons affected by one condition may stigmatize those with another. Also, health workers in leprosy, HIV, or mental health services may be stigmatized for working in such programs or for having the same condition; yet, they themselves may stigmatize the beneficiaries of the program. The model further distinguishes different types of stigma that can be recognized across conditions and cultures [3, 5, 6, 10]. Both the two perspectives and the different types of stigma have a bearing on the assessment of stigma and on selecting relevant interventions. A comprehensive definition of health-related stigma encompassing differences in perspectives and types is offered by Weiss and Ramakrishna [22], “*A social process or related personal experience characterized by exclusion, rejection, blame, or devaluation that results from experience or reasonable anticipation of an adverse social judgment about a person or group identified with a particular health problem*”.

We will demonstrate the cross-cutting nature of health-related stigma using data from studies of leprosy, HIV, TB, mental illness, inflammatory bowel disease, disability, obesity, and cancer. We will briefly present the instruments and interventions used, discuss the way they have been used across conditions, and then draw together the findings and lessons learnt regarding common aspects of stigma, proposing that ‘generic health-related stigma’ is a concept that can be used across stigmatized health conditions.

## Stigma measurement

In an attempt to ‘capture’ stigma, as well as in assessing its severity and monitoring and evaluating the impact of interventions to reduce stigma, a large number of instruments have been developed, often within specific fields such as mental health [41] and HIV [28]. In addition, tools have been developed for many of the different domains of stigma such as perceived or anticipated stigma, internalized stigma, public stigma, stigma by association, and healthcare provider-based stigma [3, 9]. For an extensive review of the types of stigma assessments as well as their use in evaluating changes in mental health-related stigma interventions, please see the recent report from the U.S. National Academy of Sciences/Institute of Medicine [42]. Unfortunately, most instruments are both condition specific and limited to a particular domain of stigma (e.g., internalized or public stigma). Despite these silos of tools, a detailed analysis of stigma assessments showed that many similarities exist in the approaches used across conditions and in the issues addressed in the items used in questionnaires and scales [3]. It is informative to pay particular attention to the instruments that have been used across several conditions, including the Social Distance Scale (SDS) [41, 43], the Berger Stigma scale [24], the Internalized Stigma of Mental Illness (ISMI) scale [44], and the Explanatory Model Interview Catalogue (EMIC) [45]. Some of these have also been used across domains to assess internalized stigma, public stigma, and healthcare provider-based stigma. Having shown applicability across different conditions, we might consider the aspects of stigma contained in these instruments to be ‘common’ elements of stigma across illnesses.

### Instruments to measure public stigma

#### Social Distance Scale (SDS)

The SDS was designed by Bogardus [46] to measure the level of acceptability of various types of social relationships between Americans and members of common ethnic groups [41, 47]. The first use of the SDS in the context of mental health was by Cumming and Cumming in 1957 [41]. The modified SDS has been widely used to measure mental health-related stigma and to understand the importance of labels attached to people with former mental illnesses [41, 48]. The modified version consists of seven questions that represent social contact with different degrees of distance, such as renting a room to someone with a condition under study, working in the same place, marrying one’s child to a person with the condition(s), or engaging someone in child care. The SDS measures the acceptability of different degrees of social distance and thus, by inference, the attitude of the respondent to the person with the condition [43]. The SDS uses gender-specific, condition-adjusted vignettes that describe a man or a woman with typical features of the condition. Seven statements with a four-option ‘degree of willingness’ scale assess the willingness of the respondent to interact with the person described in the vignette (‘Definitely willing’ (0), ‘Probably willing’ (1), ‘Probably not willing’ (2), ‘Definitely not willing’ (3)). The SDS sum score represents the attitude of the respondent towards the condition.

#### EMIC Community Stigma Scale (EMIC-CSS)

The EMIC is available in different versions. The EMIC was designed by Weiss et al. [45] to examine the nature of the illness experience, including impact of stigma, on leprosy patients in India, with special reference to their mental health. The original EMIC combined quantitative questions that were scored and qualitative, open questions that provided explanations and more depth to the quantitative scores. The instrument was designed to be usable across conditions and has since been used in a variety of conditions. The more recent studies have often only used the quantitative EMIC stigma scale, rather than the ‘mixed-methods instrument’. Later on, the instrument was adapted to assess the perception of stigmatizing attitudes and behavior among community members (lay persons), patients (affected persons) and healthcare workers [49]. The EMIC measures perceived attitude and behavior of the target group towards persons affected by the stigmatized condition. In various studies over the years, the length of the scale has varied from 8 to 25 items. The response scales contain four options, as follows: ‘Yes’ (2), ‘Possibly’ (1), ‘No’ (0), and ‘Don’t know’ (0). In the 15-item version, the sum score therefore ranges from 0 to 30. In contrast to the SDS, the EMIC-CSS asks about the views and behavior of ‘other people’ in the community, rather than that of the respondent directly. This may help to minimize social desirability bias in responses.

### Instruments to measure stigma experienced by those with the condition

#### Berger Stigma Scale

The Berger Stigma Scale was designed to measure stigma as perceived by PLHIV organized along four underlying factors, including personalized stigma (18 items); disclosure concerns (12 items); negative self-image (9 items); and concern with public attitudes about people with HIV (12 items) [24]. To develop the scale, Berger et al. [24] first developed a model of perceived stigma in PLHIV organized around precursors (perception of societal attitudes towards PLHIV and knowledge of personal sero-status), perceived stigma of having HIV (actual or potential experiences of social disqualification, limited opportunities, negative change in social identity), and possible responses to perceived stigma (change in self-image, emotional response to stigma, strategies to avoid or mitigate stigma, and redefined worldview and priorities). The actual scale items were selected and developed from a review of literature and expert consultation, field tested in the USA, and subjected to factor analysis. Responses to items are measures with a 4-point Likert scale ranging from ‘strongly agree’ to ‘strongly disagree’. While quite lengthy, the scale has since been widely used and adapted both in a range of settings and for conditions other than HIV [50–53].

#### EMIC affected persons

The aim of the EMIC developed by Weiss et al. [45] was to “*elicit illness-related perceptions, beliefs and practices in a cultural study of leprosy and mental health*”. The current ‘EMIC affected persons’ version is used to assess perceived and experienced stigma among those with the stigmatized condition. Its content is very similar to the EMIC-CSS.

#### Internalized Stigma of Mental Illness (ISMI) scale

The ISMI scale was developed to measure the subjective experience of stigma, especially the internalization of stigma [44]. ISMI subscales measure Alienation, Stereotype Endorsement, Perceived Discrimination, Social Withdrawal, and Stigma Resistance. The ISMI was developed together with people with mental illnesses. The instrument comprises 29 Likert items. Each statement is rated on a 4-point Likert scale ranging from ‘strongly disagree’ to ‘strongly agree’. The ISMI was originally validated among mental health outpatients. Results showed that the ISMI had high internal consistency and test-retest reliability. Construct validity was supported by positive correlations with measures of stigma beliefs and depressive symptoms, and negative correlations with measures of self-esteem, empowerment, and recovery orientation. More recently, a brief version of the ISMI was developed and validated [54].

## Stigma interventions

Information-based interventions are very likely the most common approach to addressing public stigma against any condition. However, they differ in content across conditions because they often address condition-specific knowledge gaps, stereotypes, fears, and other drivers of stigma; not infrequently, these are the only strategies used. However, while knowledge or education is often an essential part of stigma reduction, it is insufficient on its own [55–57].

Many authors have reviewed stigma reduction strategies and interventions from either a disease-specific or generic perspective [11, 57–63]. Evidence of effectiveness from well-designed studies using larger samples, particularly of longer-term impact, is scarce [58, 62]. However, available evidence suggests that stigma should be tackled at multiple levels, by using multiple strategies and the interventions must be context specific and continued or repeated to achieve a lasting impact [6, 8, 11, 64–66].

### Cross-condition methods to address public stigma

#### Information-based interventions

Information-based strategies are often used to reduce negative attitudes and perceived stigma in the community (public stigma). The assumption is that negative attitudes are likely to be based on a lack of knowledge, incorrect knowledge, myths, beliefs, and/or stereotypes about a given condition that can be ‘corrected’ with the right information [67]. Information-based interventions try to fill gaps in knowledge about the condition and dispel myths and demonstrate that stereotypes are often not true. An example is information about the availability of medical treatment for a given infectious disease; such information is assumed to contribute to reduction of stigma against that disease [68]. The second example is educating people with scientific facts, e.g., ‘leprosy is an infectious disease’ or ‘leprosy is caused by a bacterium’. Health promotion media campaigns have been widely used, involving printed materials, such as posters in health facilities, and/or radio and television and internet messages [69]. It is crucial that education messages and campaigns take the local worldview, culture, language, and specific fears and beliefs into account [65, 66, 70].

#### Contact between persons with the condition and the community, health professionals, or others

Facilitating contact between persons affected by a particular condition and members of the general public or healthcare workers has been shown to be effective in improving attitudes and in changing negative stereotypes [71]. This is based on the principle that attitudes can only be changed or replaced by positive attitudes when they have been shown to be dysfunctional [72]. Similarly, contact with individuals who ‘moderately disconfirm’ stereotypes is also important, i.e., with individuals who are symptomatic and are in treatment, but who also work, socialize, and have meaningful relationships [73]. The contact intervention has been used in different forms, either by facilitating direct, live contact or through electronic media. Examples are testimonies from persons affected in the community or from well-known ‘champions’, (participatory) videos and comics used during community events and meetings [74], screening on television, etc. Opportunities for discussion are also an important element.

#### Change agents/Popular opinion leaders (POLs)

Rooted in the Diffusion of Innovations Theory – a theory which focuses on how a new practice or idea can be dispersed through a social network to the point that it becomes a social norm [75] – a promising strategy to address stigmatization is the use of ‘change agents’ or POLs [76]. The hypothesis is that, when such POLs display positive attitudes, spread a non-stigmatizing message, or even fight enacted stigma in a social group, they model a new behavior and thus alter the perception and eventually even the social norm. POL interventions have been profusely and successfully applied, across different (stigmatizing) populations and across different continents, in HIV and sexually transmitted infection interventions [77–79], and more recently also in the context of the TB/HIV co-epidemic [80]. The latter on-going trial is the first attempt to apply the POL strategy to implement a cross-cutting, and thus not disease specific, stigma-reduction intervention (Rau et al., submitted for publication). Crucial to the success of such POL interventions is the selection and training of these POLs. When community members identify themselves as the members who are influential in a stratified manner, for example, by asking randomly selected respondents to nominate influential community members or by asking gatekeepers (village or organization heads) to recommend popular individuals [78], and when these potential POLs are then adequately trained, increasing knowledge as well as adapting behavior, this approach has the potential to be a suitable cross-cutting strategy applicable to a wide range of stigmatized conditions [76].

### Cross-condition methods to address stigma experienced by persons affected

#### (Peer) counselling

Peer counselling is an intervention in which suitable persons with the same condition are selected and offered training in counselling [81]; this focuses on listening and problem-solving skills, as well as increasing knowledge about the condition and, as in the case of a study in Indonesia [82], about human rights. In the case of peer counsellors, the counsellor can also serve as a role model to the counselee. Peer counselling and similar approaches have also been used in the fields of mental health and HIV, although terms like ‘peer educator’, ‘expert client’, or ‘community-linkage facilitator’ are more commonly used. However, these do not necessarily engage HIV-positive peers as educators, but rather a variety of other peers such as students in schools (e.g., Denison et al. [83]). Counselling, as part of ‘voluntary counselling and testing’ has been extensively used in HIV, but not primarily as a stigma-reduction strategy.

#### Skills building and empowerment

Interventions for socioeconomic development or improvement of the livelihoods of persons affected can be seen as economic empowerment [84, 85]. By enabling persons who are stigmatized to find a job or improve their income, self-esteem and the feeling of self-worth are improved [86]. Importantly, people get hope that there is a way out of their predicament. In low- and middle-income countries, such socioeconomic interventions are often linked to people organizing themselves in self-help groups (SHGs) [87], which may then start a saving scheme and/or be linked to a micro-finance institution (Dadun et al., submitted). Collateral-free individual or group micro-credit loans are then given from the collective savings or by the bank or institution [88]. People may start a small business or invest the loan in agricultural activities. Being able to contribute to the family income or to the community in this way often helps greatly in regaining identity and respect, either reducing public stigma or offering additional resilience to cope with it [72, 89].

## Evidence of how measurement instruments are used across conditions

Table 1 shows examples of stigma instruments that have been used across several conditions to measure attitudes and perceived and enacted stigma among the public or community. The SDS has a long history and was originally designed to assess willingness to associate with persons of different ethnic backgrounds [46]. Link et al. [90] used a version adapted for mental health to assess attitudes towards persons with mental health conditions. Lee et al. [91] assessed ‘victim blaming’ of persons with HIV or AIDS among US college students using the SDS. Peters et al. [43] used social distance as a proxy for respondent attitudes towards persons affected by leprosy in Indonesia, and a study in Germany assessed stigma against persons with obesity using the SDS [92]. The EMIC-CSS has been used across conditions most often, including in a study assessing attitudes and perceived behavior against persons with onchocerciasis [93], mental health conditions [49], Buruli ulcer [94], tuberculosis [95], and leprosy [43, 96, 97]. Additionally, the cultures were very diverse, including four countries in Africa and four in Asia.

**Table 1:** Instruments used to measure public stigma

Author	Country	Condition	Target group	*N*	Evidence of validity	Comments
SDS						
Link et al. [90]	USA	Mental health conditions	Random sample of Ohio residents	151	Alpha 0.92	
Lee et al. [91]	USA	HIV and AIDS	College students	818	No validation was reported	The SDS in this study used different items from the one adapted by Link et al. [90] used in all other studies
Peters et al. [43, 109]	Indonesia	Leprosy	Community in an endemic district	259, 213 and 375	Alpha 0.87, SDC^a^ 0.60, ICC 0.75 (95% CI 0.62–0.84); no floor or ceiling effects	
Sikorski et al. [92]	Germany	Obesity	Telephone sample of general public	1008	Alpha 0.86	
Pachankis et al. [120]	USA	44 health conditions	Experts and general public	1025	Alpha 0.84 (expert raters); 0.83 general public raters	
EMIC						
Vlassoff et al. [93]	Nigeria Cameroon, Ghana, Uganda	Onchocerciasis	Unaffected persons	410	Alpha 0.76	12-item EMIC was used
Chowdhury et al. [49]	India	Mental health conditions	Non-affected lay persons	21	Kappa 0.90 (inter-rater)	20–25 items, depending on version
Stienstra et al. [94]	Ghana	Buruli ulcer	Healthy controls	33	Alpha 0.76	15-item EMIC
Rensen et al. [96]	India	Leprosy	Non-affected community	806	Alpha 0.83; no floor or ceiling effects	13-item EMIC
Peters et al. [43, 109]	Indonesia	Leprosy	Community in an endemic district	259, 213 and 375	Alpha 0.83, SDC^a^ 0.81, ICC 0.84 (95% CI 0.75–0.90); no floor or ceiling effects	15-item EMIC
Kaehler et al. [97]	Thailand	Leprosy	Community in an endemic district	257	No validation was reported	15-item EMIC
Adhikari et al. [133]	Nepal	Leprosy	Community in an endemic district	281	No validation was reported	15-item EMIC
Sermrittirong et al. [95]	Thailand	Leprosy, tuberculosis	Community in an endemic district	236	No validation was reported	15-item EMIC

^a^SDC_group_ Smallest detectable change in the group (based on standard error of measurement (SEM), using the formula 1.96 × √2 × SEM divided √n)

*EMIC* Explanatory Model Interview Catalogue, *ICC* intraclass correlation coefficient, *SDC* smallest detectable change, *SDS* Social Distance Scale

In the same way, instruments used to assess stigma experienced by persons affected across a range of conditions are shown in Table 2. The Berger Stigma Scale, originally designed to measure perceived and experienced stigma among PLHIV [24], was successfully adapted for use in leprosy [98] and meticillin-resistant Staphylococcus aureus [53]. The ISMI was used most frequently, with no less than 81 papers covering 42 completed translations [13]. Most studies used the instrument in mental health, but other studies demonstrated the usefulness of the ISMI among persons with substance abuse, leprosy, HIV, and inflammatory bowel disease [96, 99–101]. The EMIC Affected Persons scale has been used most widely in terms of range of conditions. Originally designed to measure the impact of leprosy on the mental health of persons affected [45], it has since been used to measure experienced stigma related to mental health conditions, including depression, schizophrenia and bi-polar disorder [102–104], onchocerciasis [105], Buruli ulcer [94], HIV [101], TB [106], and leprosy [96].

**Table 2:** Instruments used to measure anticipated/perceived, internalized or experienced stigma

Author	Country	Condition	Target group	*N*	Evidence of validity	Comments
Berger stigma scale						
Berger et al. [24]	USA	HIV	People with HIV	318	Alpha 0.96; alpha sub-scales 0.90–0.93, correlation coefficient reliability 0.92. Construct validity supported by correlation with Rosenberg Self-esteem scale and Center for Epidemiological Studies-Depression scale	
Dadun et al. [98]	Indonesia	Leprosy	Persons affected by leprosy	392	Alpha 0.88; sub-scale alphas 0.79–0.84, SDC^a^ 1.37, ICC 0.75 (95% CI 0.64–0.83); no floor or ceiling effects; construct validity supported by correlation with the P-scale and WHOQOL-BREF	Renamed ‘SARI Stigma Scale’ because of substantial changes to structure
Rump et al. [53]	Netherlands	MRSA	MRSA carriers	57	Validity was supported by correlation with the RAND mental health inquiry	An adapted version was used
ISMI						
Boyd Ritsher et al. [44]	USA	Mental health conditions	Mental health outpatients	127	Alpha 0.90 (sub-scales 0.58–0.80); test-retest reliability *r* = 0.92 (*n* = 16) (sub-scales 0.68–0.94); good construct validity	29 items
Brohan et al. [134]	13 European countries	Bipolar disorder and depression	Mental health patients	1182	Alpha 0.94; construct validity supported by strong correlations with an empowerment scale and a devaluation and discrimination scale	24-item ISMI was used, excluding the Resilience sub-scale
Singh et al. [135]	India	Mental health	Persons with severe mental disorders	161	Alpha 0.86; ICC test-retest reliability (*n* = 31) sub-scales range 0.84–0.96; 5-component structure supported by factor analysis; good correlation with EMIC	
Luoma et al. [99]	USA	Substance abuse	Adults with a substance use disorder	88	Alpha 0.82 and 0.92 at pre- and post-assessment	
Stevelink et al. [101]	India	HIV Leprosy	Patients/persons affected	95 HIV 95 leprosy	Alpha 0.87 Alpha 0.91	
Rensen et al. [96]	India	Leprosy	Affected persons	806	Alpha 0.96; sub-scale alphas 0.79–0.96; weighted kappa 0.62 (*n* = 49); no floor or ceiling effects	18-item ISMI
Taft et al. [100]	USA	Inflammatory bowel disease	Irritable bowel disease patients	191	No validation was reported	
Arachchi et al. [136]	Sri Lanka	Leprosy	Affected persons	132	No validation was reported	
EMIC affected persons						
Weiss et al. [45]	India	Leprosy, mental health	Patients	56 + 31 controls	Item-wise kappa values 0.62–0.93 (*n* = 16–18); association with established mental health instruments (SCID and HDARS)^b^supported construct validity	8-item EMIC
Raguram et al. [102, 103]	India	Depression, schizophrenia	Patients Family, caretakers	80 80	Alpha 0.71 Alpha 0.81	10-item EMIC 13-item EMIC
Brieger et al. [105]	Nigeria	Onchocerciasis	Patients	500	No validation was reported	13-item EMIC was used
Vlassoff et al. [93]	Nigeria Cameroon Ghana Uganda	Onchocerciasis	Patients	469	Alpha 0.80	13-item EMIC was used
Chowdhury et al. [49]	India	Mental health conditions	Patients	25	Kappa 0.89 (inter-rater)	20-25 items, depending on version
Stienstra et al. [94]	Ghana	Buruli ulcer	Patients	33	Alpha 0.65 (of 11 items asked of both patients and controls)	15-item EMIC
Weiss et al. [106]	Bangladesh India Malawi Colombia	Tuberculosis	Patients	102 127 100 98	Alpha 0.77 Alpha 0.85 Alpha 0.63 Alpha 0.65	18-item EMIC
Stevelink et al. [101]	India	HIV Leprosy	Patients/persons affected	95 HIV 95 leprosy	Alpha 0.76 Alpha 0.83	
Rensen et al. [96]	India	Leprosy	Patients	806	Alpha 0.88; weighted kappa 0.70; no floor or ceiling effects	17-item EMIC
Grover et al. [104]	India	Bi-polar disorder	Patients	185	Alpha 0.94; good correlation with ISMI and Participation scale scores	15-item EMIC
Arachchi et al. [136]	Sri Lanka	Leprosy	Patients	132	No validation was reported	Not reported

^a^SDC_group_ Smallest detectable change in the group (based on standard error of measurement (SEM), using the formula 1.96 × √2 × SEM divided √n) *ICC* intraclass correlation coefficient, *ISMI* Internalized Stigma of Mental Illness, *MRSA* Meticillin-resistant Staphylococcus aureus, *P-scale* Participation Scale, *WHOQOL-BREF* WHO Quality of Life scale – Brief

^b^*SCID* Structured Clinical Interview for DSM-III-R; *HDARS* Hamilton Depression and Anxiety Rating Scale,

### Evidence of how stigma interventions are used across conditions

Interventions to reduce public stigma were also very similar across diverse conditions.

Table 3 shows examples of information-based interventions being used to address attitudes of college students towards persons with mental health conditions in the USA [107], general public attitudes towards HIV in Ghana [108], and community attitudes to leprosy in Indonesia [109]. Another very commonly used stigma intervention is the contact intervention, which was used with success to improve attitudes to mental illness among college students in the USA [110], attitudes towards PLHIV among nurses in Hong Kong [111], and attitudes of community members towards persons affected by leprosy in Indonesia [74, 109]. Education about the condition and related beliefs and fears, and contact between persons with the concerned conditions and members of the community or other target group are often used together; this combination of interventions has been shown to work across conditions and cultures [11, 60, 62, 109, 111, 112]. Training and engagement of POLs or change agents was successful in different conditions (leprosy, HIV, and TB) and very different cultural settings (Nepal, USA, Peru, China, and South Africa) [77, 78, 113, 114].

**Table 3:** Interventions used across conditions to address public stigma (attitudes and behavior)

Author	Country	Condition	Target group	*N*	Evidence of effectiveness	Comments
Information-based approaches						
Masuda et al. [107]	USA	Psychological disorders	College students	95 (43 + 52)	The CAMI scores for the educational workshop lowered at post-intervention and 1-month follow-up among participants with higher levels of psychological flexibility (scored 67 or higher on the Acceptance and Action Questionnaire)	CAMI administered at beginning and end of workshop, and at 1-month follow-up
Boulay et al. [108]	Ghana	HIV	General public	2746, 2926	Attitudes related to a punitive response to PLHA both improved over time and were positively associated with exposure to the program’s campaign; overall, respondents exposed to the campaign were 45% more likely than those not exposed to be willing to care for a HIV-infected relative, and 43% more likely to believe that an HIV-infected female teacher should be allowed to continue teaching	
Peters et al. [109]	Indonesia	Leprosy	Community in an endemic district	213 and 375	Knowledge about leprosy increased and that negative attitudes reduced significantly; at baseline, 87% considered leprosy curable and 31% thought leprosy was still contagious after treatment; this had improved after the contact event to 98% and 7%, respectively	Post-intervention result measured after 3 months
Contact						
Peters et al. [109]	Indonesia	Leprosy	Community in an endemic district	213 and 375	The EMIC and SDS stigma scores reduced both among those attending ‘contact events’ (effect sizes 0.75 and 0.81, respectively) and in the wider community (effect size 0.47 and 0.54)	Contact was through testimonies on video plus a live testimony given at ‘contact events’ with community groups; post-intervention results were measured on average 1–1.5 years after the contact events
Corrigan et al. [110]	USA	Mental illness	College students	257	Participants in the contact intervention group showed significant reduction in avoidance and segregation factors with the Attribution Questionnaire at post-intervention and 1-week follow-up; participants in contact condition also showed significant reduction in pity and improvement in power from pre- to post-intervention	Contact through video was used; measures administered pre-test, post-test, 1-week follow-up
Paxton [137]	?	HIV	Young people	1230	HIV-positive speakers were effective in decreasing fear and stigmatization among the audience; meeting HIV-positive people decreased fear and prejudice, reinforced messages about protective behavior and increased the belief that HIV is preventable; the improved attitudes remained significant over 3 months	
Uys et al. [71]	Lesotho, Malawi, South Africa, Swaziland, Tanzania	HIV	Nurses PLHA	41 PLHA 134 nurses	PLHA involved in the intervention teams reported less stigma and increased self-esteem; nurses in the intervention teams and those in the target group reported no reduction in stigma or increases in self-esteem and self-efficacy, but their HIV testing behavior increased significantly	A pre- and post-test was done to measure stigma, self-esteem and self-efficacy; the post-test was conducted within 1 month after the intervention
Yiu et al. [111]	Hong Kong	HIV	Nursing students	89	In both the knowledge-only group and the knowledge-contact group, significant improvement in AIDS knowledge, stigmatizing attitudes, fear of contagion, willingness to treat, and negative affect were found at post-test; the effects on AIDS knowledge, fear of contagion, willingness to treat, and negative affect were sustained at follow-up for both groups Intergroup comparisons at post-test showed that the effectiveness of the knowledge-contact program was significantly greater than the knowledge program in improving stigmatizing attitudes; no significant difference between the two groups was found at follow-up	
Change agents/ Popular opinion leaders						
Kelly et al. [114]	USA	HIV	Gay men	8 cities	In the four intervention cities a statistically significant reduction was found in the mean frequency of unprotected anal intercourse during the previous 2 months and a significant increase in the mean percentage of occasions of anal intercourse protected by condoms	
Cross & Choudhury [76]	Nepal	Leprosy	Community	152 SHG participants	The Stigma Elimination Programme had a significant impact at community level and is recognized as a positive force by district level officials of Her Majesty’s Government of Nepal; as direct effects of SHG activity, 1060 people have had some basic education, many people now have access to clean water, some have the benefits of improved sanitation and others have improved physical access to amenities, over 200 people are now generating income from their own micro enterprises	
Young et al. [77]	Peru	HIV	Community	1327 POL, 1722 comparison	HIV-related stigma significantly reduced from baseline to 12-month follow-up and from baseline to 24-month follow-up among participants in the POL intervention	5 stigma items assessed at baseline, 12-month, and 24-month follow-up
Li et al. [78]	China	HIV	Healthcare workers	1750 POL	Reduced prejudicial attitudes (estimated difference = – 2.40; *p* < 0.001), reduced avoidance intent towards people living with HIV (estimated difference = – 1.10; *p* < 0.001), and increased institutional support in the hospitals (estimated difference = 0.39; *p* = 0.003) at 6 months after controlling for service providers’ background factors and clinic-level characteristics	The intervention effects (6 months) were sustained and strengthened at 12 months

*CAMI* Community Attitudes toward the Mentally Ill, *EMIC* Explanatory Model Interview Catalogue, *POL* popular opinion leaders, *PLHA* people living with HIV and AIDS, *SDS* Social Distance Scale, *SHG* self-help group

Interventions to mitigate the impact of stigma have addressed the mental wellbeing of the persons affected, their resilience, self-efficacy and sense of self-worth, and ability to speak up for themselves through empowerment, skills building, and participation in the actual interventions. Nuwaha et al. [115] and Jürgensen et al. [116] found home-based counselling to be successful in reducing different aspects of HIV-related stigma in Uganda and Zambia. Conner et al. [117] found peer education was effective to reduce internalized stigma in a small study with older adults with mental health conditions in the USA. Across the globe, Lusli et al. [82] trained lay and peer counsellors among persons affected by leprosy in Cirebon, Indonesia; they, in turn, counselled others. Their approach, which included building resilience, restoring dignity, and awareness of human rights, was shown to be effective in reducing stigma, improving social participation, and improving quality of life among the counselees [118].

Skills building and empowerment of persons who are stigmatized is another strategy shown to be effective across conditions and cultures. The Stigma Elimination Project in south Nepal trained a small group of persons with visible signs of leprosy who showed leadership potential [76], who became leaders of a rapidly growing number of SHGs. After 3 years, the level of social participation of SHG members was at the level or better than that of a community control group. Bellamy and Mowbray [119] found a ‘supported education program’ to be successful in empowering adults with mental health conditions in the USA and strengthening their self-efficacy to (re-)enter post-secondary education. Dalal [72] reported empowerment of persons with disabilities in north India to be very successful in overcoming shame, increasing social participation, and improving health outcomes as well as in changing community attitudes towards disability. Uys et al. [71] used skills building and empowerment among both nurses and PLHIV to reduce stigma and improve quality of care in healthcare settings in five African countries. This was successful in reducing stigma and increasing self-esteem among PLHIV, but did not affect stigma among the nurses. However, the HIV testing behavior of the latter improved significantly.

## The concept of health-related stigma

The current paper demonstrates that ‘health-related stigma’ is a viable concept with clearly identifiable characteristics that are similar across a variety of stigmatized health conditions in very diverse cultures. The etiology of stigma differs between conditions and sometimes between cultural settings. For example, persons with schizophrenia are stigmatized because people perceive them to be unpredictable or dangerous, while PLHIV may be stigmatized and discriminated against because, in certain cultures, HIV is associated with homosexuality and promiscuity, and because it is perceived to be a highly infectious, as well as fatal and incurable disease. Leprosy is often stigmatized because of the notion that the person affected has committed a sin or broken a taboo, either in this or a previous life; it may also be due to fear of the associated disfigurements. Even regarding the etiology and origins of stigma and discrimination, ‘shared dimensional features’ can be readily recognized. Pachankis et al. [120] used the six features identified by Jones et al. [1] (aesthetics, concealability, course, disruptiveness, origin, and peril) as a taxonomy for characterizing and investigating the perceived burden of stigma on health and wellbeing across no less than 93 health and other conditions.

As noted in the Background section, the expressions or manifestations and psychosocial consequences of stigma and discrimination are often remarkably similar, even across very different cultures and levels of socioeconomic development [3, 5, 6, 8]. Stigma starts when salient differences between people are recognized, labelled, and connected to stereotypes or social identities [16]. This process leads on to a separation between ‘us’ and ‘them’, resulting in status loss and discrimination. Depending on the culture and time, these differences may include a large variety of characteristics, including ethnicity, sexual orientation, skin color, body weight, religious beliefs, and a wide range of health conditions. In this paper, we limited ourselves to health conditions, though we are well aware of the intersectionality of stigma where health-related and other stigmas interacted and may compound each other [121–123]. A substantial body of literature addresses the intersectionality of stigma related to particular conditions. For example, Lowie et al. [121] examined how gender, race, sexual orientation, and sex work intersect with HIV-related stigma. Very few studies have investigated types of stigma, stigma assessment, or stigma interventions across multiple stigmatized conditions. A notable exception are the studies that have looked jointly at HIV- and TB-related stigma [124, 125]. Mak et al. [126] compared SARS-related stigma with that of HIV and TB. However, the great majority of studies of stigma related to health conditions occurred within the specific field dealing with a specific condition or range of conditions (e.g., mental health conditions). Within these fields, authors have demonstrated the similarities and differences across cultures and languages, e.g., in leprosy [127], HIV [8], TB [106], and mental health [6]. However, very few studies have attempted in-depth analyses across different health conditions. Van Brakel [3] included mental health, epilepsy, HIV, leprosy, TB, Buruli ulcer, onchocerciasis, and physical disability in his review of measurement of health-related stigma, noting many commonalities in the approaches and tools used to measure different stigmas. A more recent review investigated stigma across 10 neglected tropical diseases and noted many similarities in the types of stigma reported, the manifestations, and the approaches used to mitigate stigma [10]. Although not limited to health-related stigma, the study of Pachankis et al. [120] included 44 health conditions. They examined similarities and differences regarding each of the six characteristics proposed by Jones et al. [1] and investigated their association with a range of different stigma-related measures, including the SDS. One of the findings was that “*Visibility and course were not associated with social distance. In contrast, participants indicated a desire for greater social distance with respect to stigmatized statuses that were perceived as disruptive, aesthetically unappealing, onset controllable, and perilous*” [120]; these features are shared by many stigmatized health conditions.

The above findings show that there is a scientific rationale for the concept of health-related stigma, as proposed by Weiss et al. [19] and Scambler [20, 128]. A more generic approach to the study of health-related stigma opens up important practical opportunities. This paper illustrated this with two aspects of work – stigma measurement and interventions to reduce or mitigate stigma.

## Towards common stigma measurement approaches for health-related stigma

If it were possible to measure stigma and discrimination using generic instruments, this would have clear advantages, especially for use in public health programs and social services. Use of measurement tools requires training. With a different tool for each condition, staff in health and social services have to learn and keep up with many different instruments, some of which they may only use infrequently, thus never acquiring a ‘feel’ for the instrument and the results it produces. In the current age of mobile data collection, one could envisage that adaptation of a given instrument to a particular condition would be done by just indicating on the opening screen which condition one wants to test; the software would automatically adapt the instrument to that condition. Tools for which this would be very easy are those indicated in Table 1 and Table 2. Instruments like the SDS, EMIC, and ISMI were shown to be highly suitable for use across conditions since the content includes manifestations and impact common to many stigmatized health conditions.

Researchers in the health-related stigma field can clearly also benefit from the use of instruments that can be adapted very easily for use across conditions; the study of Pachankis et al. [120] illustrates this point very nicely.

A disadvantage of using generic instruments is a potential lack of sensitivity and/or specificity. Where this would be required, one could envisage using an add-on module comprising a few condition-specific items. This would retain the advantage of a common core of items that can be used and compared across conditions. A very similar approach that is widely accepted is the measurement of health-related quality of life. Generic tools like the WHO Quality of Life scale, abbreviated version (WHOQOL-BREF), and the Short Form 36 items are used across a myriad of disabling and stigmatized conditions and in very culturally diverse circumstances. In certain situations, add-on modules are used, such as the WHOQOL-DIS for disability, or the WHOQOL-SRPB for the effects of spirituality, religion and personal beliefs.

## Towards common stigma intervention approaches for health-related stigma

Many of the same advantages that apply to cross-condition measurement tools also apply to interventions.

Table 3 and Table 4 illustrate the several interventions that have already been used successfully with multiple conditions; this is hardly surprising because of the common social and psychological processes underlying health-related stigma [5, 16, 19]. Manifestations, such as difficulties in finding and maintaining employment, broken relationships, and impacts on socioeconomic status and mental wellbeing, including shame and reduced self-esteem, are common across conditions, thus offering entry points for cross-cutting interventions. It should be noted that, although the studies included have been classified under one, or at the most two, intervention types, almost all studies used multiple interventions. Sometimes, these addressed different levels and sometimes they addressed both the sources of stigma and the persons affected by stigma. Even when used on a single level, there is evidence that using multiple interventions is more effective than using a single intervention [111].

**Table 4:** Interventions used across conditions to address internalized, anticipated, or experienced stigma or disclosure concerns among persons with the condition

Author	Country	Condition	Target group	*N*	Evidence of effectiveness	Comments
(Peer) counselling/education						
Nuwaha et al. [115]	Uganda	HIV	Adults in the community	1402 before; 1562 after	The proportion of people who had ever tested for HIV increased from 18.6% to 62% (*p* < 0.001). Among people who had ever tested, the proportion who disclosed their HIV test result to a sexual partner increased from 41% to 57% (*p* < 0.001). The proportion who wanted the infection status of a family member not to be revealed decreased from 68% to 57% (*p* < 0.001)	This concerned a home-based counselling and testing program
Jürgensen et al. [116]	Zambia	HIV	Adult 16 and above	1500 pre 1107 post	There was an overall reduction of 7% in stigma from baseline to follow-up, mainly due to a reduction in individual stigmatizing attitudes but not in perceived stigma; the reduction did not differ between the trial arms (*p* = 0.423) Being tested for HIV was associated with a reduction in stigma (*p* = 0.030) and HBVCT had a larger impact on stigma than other testing approaches (*p* = 0.080 vs. *p* = 0.551)	HBVCT trial
Conner et al. [117]	USA	Mental illness	Older adults with depression in community	19	ISMI scores significantly reduced after participating in the 3-month peer educator intervention	
Lusli et al. [82]	Indonesia	Leprosy	Persons affected by leprosy	67 clients; 57 controls	Significant reduction was observed between the before and after total SARI Stigma scale scores (mean difference clients 9.6 vs. 5.6 for controls), Participation scale scores (mean difference clients 3.7 vs. 1.4 for controls) and WHOQOL-BREF scores (mean difference clients +6.5 vs. – 2.0 for controls)	Outcome assessed on average 1–1.5 years after baseline
Skills building and empowerment						
Cross & Choudhary [76]	Nepal	Leprosy	Persons affected by leprosy	152 SHG participants and 102 controls	Social participation in the intervention group (where participants were working as change agents) was much better than in the control group; the median scores on the Participation scale were 0 and 7, respectively (*p* < 0.0001, Kruskal–Wallis test)	
Bellamy & Mowbray [119]	USA	Mental health conditions	Adults with mental illness	397	After a 6-month follow-up, those with greater participation showed greater quality of life, empowerment, school/vocational enrollment, and encouragement from mental health workers; a significant condition effect was found for empowerment (*p* < 0.01) and for school efficacy (*p* < 0.05); at 12-month follow-up, college or vocational enrollment had increased significantly	
Dalal [72]	India	Disabilities	Persons with disabilities		The project resulted in four types of outcomes: (1) increased visibility and participation of people with disabilities in community activities; many of them stepped out of their houses for the first time; (2) the number of physically challenged attending meetings gradually increased from none to 30–40% during the 3 years; (3) there was almost a 150% increase in immunization against polio in the third year; (4) a greater number of people were reaching out to hospitals and rehabilitation centers in a nearby city; people who earlier thought that nothing could be done were now exploring the possibilities of medical rehabilitation with community support	
Uys et al. [71]	Lesotho, Malawi, South Africa, Swaziland, Tanzania	HIV	Nurses PLHA	41 PLHA 134 nurses	PLHA involved in the intervention teams reported less stigma and increased self-esteem Nurses in the intervention teams and those in the target group reported no reduction in stigma or increases in self-esteem and self-efficacy, but their HIV testing behavior increased significantly	Pre- and post-test measured stigma, self-esteem and self-efficacy; the post-test was conducted within 1 month after the intervention
Dadun et al. [85] Dadun et al. (submitted)	Indonesia	Leprosy	Persons affected by leprosy	20 qualitative + 30 quantitative	In qualitative interviews, clients reported growing businesses, better self-esteem, improved interaction with neighbor and most also less stigma than before; in some cases, disclosure concern remained high; in the quantitative interviews, the mean difference between the pre- and post-assessment total score of the SARI Stigma scale for socioeconomic development clients and the control group was 10.0 vs. 6.7, for the Participation scale 3.6 vs. 1.4 and for the WHOQOL-BREF + 4.32 vs. – 2.00	Outcome assessed on average 1–1.5 years after baseline

*ISMI* Internalized Stigma of Mental Illness, *HBVCT* home-based counselling and testing, *PLHA* people living with HIV and AIDS, *SHG* self-help group, *WHOQOL-BREF* WHO Quality of Life scale – Brief

In contrast to the use of instruments, certain interventions can even be used across multiple conditions simultaneously. This is the case for counselling services, skills-building, and economic empowerment programs and SHGs, for example.

One major problem is that funders of stigma reduction programs usually only fund condition-specific studies, measures, and interventions. Surveillance for stigma and stigma-mitigating interventions can be integrated in regular health and social services using generic tools and interventions. For example, in China, a stigma-reduction intervention focused on infection control through education and providing adequate supplies for practicing universal precautions [78, 129]. Similarly, in Vietnam, a stigma-reduction intervention allowed healthcare facility staff to develop practical skills around infection prevention and a code of practice, tailored for their own hospital’s needs, on implementing stigma-free practices and universal precautions [130]. In the field of leprosy, counselling to mitigate the effects of stigma has been integrated in a range of hospitals that offer leprosy services in Nepal and India [131, 132].

Using generic tools and interventions within the health services would help overcome the siloed approach by demonstrating the advantages of integration, while simultaneously contributing to health systems strengthening. Dr Gottfried Hirnschall, WHO HIV Director, said, “*We need to ensure that frontline health workers have the information and skills required to effectively identify, address and avoid stigma and discrimination of all types, including those related to HIV*”.^1^ Developing generic health-related stigma assessment and monitoring tools as well as generic stigma interventions would provide essential building blocks for making this possible.

## Limitations

A limitation of this paper is that it is not based on a systematic literature review. We can therefore make no claim to completeness of the evidence to support the concept of health-related stigma. However, we believe that the cross-condition use of each instrument and intervention has been adequately demonstrated through our use of these selective, illustrative examples.

## Conclusions

- Researchers, research funders, public health and social services managers, and health and social services practitioners should adopt cross-cutting, more cost-effective approaches to health-related stigma, seeking to use generic instruments and interventions where possible.
- Stigma studies should demonstrate how stigma theory and frameworks apply across conditions and delineate commonalities, as well as condition-specific exceptions that might be important for understanding, measurement, or interventions.
- Researchers studying stigma should approach the issues more generically, adapting (potentially) generic stigma instruments to containing an optimal common core of items, identifying, where necessary, condition-specific add-on items or modules.
- Stigma studies should be commissioned to demonstrate the advantages and effectiveness of cross-condition approaches to measurement and interventions.

## Abbreviations

CSS: Community Stigma Scale
EMIC: Explanatory Model Interview Catalogue
ISMI: Internalized Stigma of Mental Illness
PLHIV: People living with HIV
POLs: Popular opinion leaders
SDS: Social Distance Scale
SHG: Self-help group
TB: Tuberculosis
WHOQOL-BREF: WHO Quality of Life scale

## Footnotes

1 http://www.who.int/mediacentre/commentaries/zero-discrimination-day/en/; Accessed 13 June 2018
